# Efficacy of Levamisole in children with Frequent Relapsing and Steroid Dependent Nephrotic Syndrome at Tertiary Care Center-Karachi

**DOI:** 10.12669/pjms.36.6.2337

**Published:** 2020

**Authors:** Khemchand N Moorani, Aasia Mohammad Zubair, Nanga Ram Veerwani, Harnam Jaichand Hotchandani

**Affiliations:** 1 Khemchand N Moorani, FCPS, MCPS, MBBS. Professor of Pediatric Nephrology, Department of Pediatric Nephrology, The Kidney Center Postgraduate Training Institute, Karachi Sindh, Pakistan, Department of Pediatric Nephrology, National Institute of Child Health, JSMU, Karachi, Pakistan; 2 Aasia Mohammad Zubair, FCPS, MBBS. Medical Officer, Department of Pediatric Nephrology, The Kidney Center Postgraduate Training Institute, Karachi Sindh, Pakistan, Department of Pediatric Nephrology, National Institute of Child Health, JSMU, Karachi, Pakistan; 3 Nanga Ram Veerwani, FCPS, MBBS. Medical Officer, Department of Pediatric Nephrology, The Kidney Center Postgraduate Training Institute, Karachi Sindh, Pakistan; 4 Harnam Jaichand Hotchandani, MBBS. Senior Medical Officer, Department of Pediatric Nephrology, The Kidney Center Postgraduate Training Institute, Karachi Sindh, Pakistan

**Keywords:** Remission of proteinuria, Frequent relapsing, Steroid dependent nephrotic syndrome, Relapses, Levamisole

## Abstract

**Objectives:**

To determine the effectiveness of levamisole in maintaining remission of proteinuria in children with frequent relapsing and steroid dependent nephrotic syndrome (FR/SDNS).

**Methods:**

This observational study on 81 children with FR /SDNS was carried out from June 2007 - June 2017 at The Kidney Center-Postgraduate Training Institute, Karachi-Pakistan. Levamisole (leva) along with low dose prednisolone on alternate day (AD) was used after induction of remission with daily oral prednisolone in children with FR/ SDNS for 6-36 months. Patients with steroid resistance were excluded. Data was analyzed using descriptive statistics.

**Results:**

Eighty-one patients with FR (66) or SD (15) received levamisole treatment. Mean age at diagnosis was 3.72 ±2.33 years. Levamisole was used on AD in 59.25% and daily in 40.74% of cases. Twenty-four could not complete six months and were excluded, 57 patients completed treatment duration of 15.68±9.93 months and 51 post-leva follow-up of 11.70±11.23 months. Mean leva-dose was 1.73±0.67 mg/kg/ patient. Mean cumulative prednisolone dose per patient before, on-leva and post-leva was 3389.81±2785.22, 2471.97±2024.98 and 661.37± 905.37 mg respectively. Mean relapse rate per year before leva, on -leva and post -leva was 3.30 ±0.50,0.98± 1.1and 0.79±1.27 respectively. Levamisole was effective in 90% of patients. During post-leva follow up, 76.4% patients, maintained remission, whereas 23.5% behaved as FR/SD and require further immunosuppressive therapy.

**Conclusions:**

Levamisole was effective in maintaining remission in 90% while on treatment, whereas it maintained remission after discontinuation in 76.4% cases. Levamisole may be used as first steroid sparing agent before other immunosuppressive therapies in children with FR/SDNS. Further studies are required for optimal duration and dosage schedule.

## INTRODUCTION

Majority of children with nephrotic syndrome are steroid sensitive minimal change disease(MCD), but after initial response to standard corticosteroid therapy, more than 80% experience relapses.^1^ Among the steroid sensitive children, 40-60 % experience frequent relapses (FR) and 30-50% of MCD become steroid dependent (SDNS).^1^ Patients with FR and SD who require repeated courses of steroid are at risk of long -term steroid toxicities and serious infections, thus may affect the qualities of life.^2^ In children with FRNS, steroids can be stopped intermittently but not in SDNS and patients with SD are more vulnerable to steroid toxicity and require aggressive immunosuppressive protocols.^1^,^3^

Management strategies for children with FR and SDNS, include prolonged use of low dose oral prednisolone (OP) on alternate day (AD), levamisole along with AD low dose OP, cyclophosphamide (CPM) for 2-3 months, mycophenolate mofetil (MMF) and calcineurin inhibitors (CNIs) like cyclosporine or tacrolimus.^4^-^6^ Rituximab (anti-CD20) has been used in developed countries for difficult cases of FRNS /SDNS.^7^

Minimal change nephrotic syndrome is a T-cell disorder with type 2 immune response in active disease state and levamisole exerts an immunomodulatory effect, upregulating Th1 and CD4 T cells through activation of interleukin-18 and interferon- gamma respectively.^8^ There are studies published locally on various aspects of nephrotic syndrome but only one on efficacy of levamisole in children with FR/SDNS. 9-11

We in this study report our experience of levamisole as the first-line alternative steroid sparing agent in FRNS and SDNS. Objective of the study was to determine the effectiveness of levamisole in maintaining remission of proteinuria in children with FR and SDNS.

## METHODS

This observational study comprises of 81 children with either FR or SDNS who received levamisole over 10 years from June 2007 - June 2017 at The Kidney Center-Postgraduate Training Institute, Karachi-Pakistan. Institutional Ethical Review Board approval (No.91-NEPH-012020(EXEMPTION) was taken. Formal consent from parent or patients was taken before starting treatment with levamisole. Convenient sampling technique was used for this study. Initial episode of NS was treated with daily OP (60mg/m^2^) for 4-8 weeks followed by 40mg/m^2^ on AD for 12-24 weeks. Based on subsequent number and frequency of relapses, patients were categorized as FR if >2 relapses in 6 months or > 4 in a 12 months’ period and SDNS if child develops two consecutive relapses on steroid therapy or within 14 days of switching to AD prednisolone.^12^

Levamisole was used for 6-36 moths in a dose of 2-2.5 mg/ kg/day either daily or AD along with low dose OP (0.25-0.5 mg/kg dose) on AD after induction of remission with daily OP for relapse. Patients were followed initially at one month with spot urine protein creatinine ratio (spot Ur PCR) and complete blood count, subsequently 2-3 monthly with tapering of AD prednisolone from 1.5 mg/kg to 1 mg/kg at one month then 0.5 mg/kg on alternate day for first 3-6 months and then 0.25mg/kg on alternate day. Children who were not able to complete first six months due to non-compliance to levamisole or those whose parents opted for alternate immunosuppressive therapy were excluded from final analysis.

Patients with initial steroid resistance were also excluded. Data including patient’s age, body surface area (BSA), height, weight, indications (FR/SD), dose of levamisole, number of relapses and cumulative dose of OP before, on-leva and post- leva treatment were collected on predesigned proforma and analyzed using descriptive statistics on SPSS version 16. Qualitative variables like gender, type of NS and treatment outcome were represented by frequencies and percentages whereas quantitative variables like age were represented by mean ± standard deviation. Student-T -test was applied to compare the relapse rate and cumulative steroid dose before, on-leva and post -leva follow-up. P -value less than 0.05 was considered as significant.

## RESULTS

There were 81 (62.32%) out of 130 children with SSNS who received levamisole during the study period. The characteristics of children who received leva during study period are shown in Table-I. Among 81 patients, 66 (81.48%) were FR and 15 (18.51%) were SDNS. This table also shows that the mean age of study participants at initial diagnosis and at time of analysis was 3.72 ±2.33 and 8.44±3.70 years respectively.

**Table-I:** Characteristics of patients on Levamisole Treatment in Frequent Relapsing and Steroid Dependent Nephrotic Syndrome. n=81.

Variable	N (%)/ mean ±SD
***Gender***	
Male	48(59.3%)
Female	33(40.7%)
Age at diagnosis (years)	3.72 ±2.33
Present age (years)	8.44±3.70
Present weight (kg)	25.18±11.27
Present height (cm)	118.11±20.25
Present BSA (msq)	0.88±0.27
***Indications of levamisole***	
FRNS	66(81.4%)
SDNS	15(18.5%
Levamisole dose (mg/kg/day)	1.74±0.68
Duration of levamisole (months)	15.68±9.39
Post-leva follow up period(months)	11.70±11.23
***Cumulative steroid dose(mg)***	
Pre-leva	3389.81±2785.22
On-leva	2471.97±2024.98
Post-leva	661.37± 905.37
***Relapse rate per year***	
Pre-leva	3.30 ±0.50
On-leva	0.98± 1.1
Post-leva	0.79±1.27

Levamisole was used on AD dosage in 48 (59.25%) patients during early years and daily dose in 33 (40.74%) cases during later years. Among 81 enrolled patients, 24 could not complete minimum duration of six months so excluded, 57 patients completed leva- treatment of 15.68±9.93 months and 51 cases were followed for 11.70±11.23 months after discontinuation of leva (Table-I).

The mean dose of levamisole used per patient was 1.73±0.67 mg/kg and mean duration of leva- treatment was 15.68±9.93 months. This table also reveals that the mean cumulative doe of prednisolone per patient before treatment with leva (12 months), on -leva (15.68±9.93 months) and during post -leva follow up was 3389.81±2785.22,2471.97±2024.98 and 661.37± 905.37 respectively. Mean relapse rate per year before leva, on -leva and post -leva was 3.30±0.50,0.98± 1.1 and 0.79±1.27 respectively.

Relapse rate decreased significantly (p=0.001) with the use of levamisole in children with FR/SD and reduction in cumulative steroid dose from 3389.81±2785.22 mg before leva to2471.97±2024.98 mg while on treatment and to 661.37± 905.37 mg during post-leva treatment period. However, there was no significant reduction in cumulative steroid dose as shown in Table-II.

**Table-II:** Cumulative steroid dose and relapse rate in children with Frequent Relapsing and Steroid Dependent Nephrotic Syndrome.

Duration (months)	Cumulative dose of steroid (mg/patient) Mean±SD**	Number of relapses per patient Mean±SD	P value*
Pre-Leva (12 months) n=81	3389.81±2785.22	3.30 ±0.50	0.001
On- leva (15.68±9.93 months) n=57	2471.97±2024.98	0.98± 1.1	
Post-Leva (12 months) n=51	661.37± 905.37	0.79±1.27	

* Significant for relapse rate (pre, on and post leva relapse rate).

** Not significant for cumulative dose.

Outcome of children treated with levamisole is shown in Table-III. There was no relapse or had less than two relapses on -leva in 46(90.1%) and considered as effective whereas 11(19.2%) had >2 relapses in six months and declared as not effective. During post-leva follow-up period of 11.70±11.23 months, 39(76.4%) patients out of 51 maintained remission whereas, 12(23.5% behaved as FR/SD and require further immunosuppressive therapy. There was pancytopenia and allergic rashes each in one child.

**Table-III:** Outcome of levamisole in children with frequent relapsing and steroid dependent nephrotic syndrome.

On -levamisole outcome (N=57)	
Effective	46(90.1%)
Not effective	11(19.2%)
Post- levamisole outcome (N=51)	
Maintained remission	39(76.4%)
Frequent relapsing/steroid dependent	12(23.5%

## DISCUSSION

Management of children with steroid sensitive nephrotic syndrome who behave as FR or SD is challenging and various immunosuppressant agents have been evolved during last two decades,^1^,^3^-^5^ Options of sequential immunosuppressive therapy for children with FR /SD include use of low dose OP on AD, levamisole, cyclophophosphamide, CNIs (cyclosporin and tacrolimus), mycophenolate mofiteil and more recently rituximab.^3^-^7^ Currently, there is still no consensus on whether one immunosuppressive agent is more effective than other.^1^,^3^,^4^

Levamisole is an immunomodulant imidazothiol-derived anthelminthic agent and has been the center of current research in FR and SDNS.^13^,^14^ The efficacy and safety of levamisole has been documented in many observation studies and in randomized controlled trials (RCTs) from many European and Asian countries including Pakistan.^6^,^10^,^14^-^16^

We recently published data on experience of immunosuppressive agents in primary nephrotic syndrome at tertiary care center but current study aimed at determining the effectiveness of using levamisole in children with FR and SD.^12^

It was found that use of leva in children with FR and SDNS was effective in maintaining relapse free remission over 12 months while on treatment and more than 12 months after discontinuation of levamisole (Table III and IV). Levamisole use resulted in significant reduction of relapse rate per patient, from 3.30±0.50 to 0.98± 1.1 on levamisole and even after discontinuation of levamisole to 0.79±1.27 (p=0.001). (Table-III). Similar response with use of leva as steroid sparing agent in FR and SDNS have been reported in many studies.^15^-^18^

**Table-IV:** Comparison of our study results with an Indian Study.^17^

Characteristics	Our study(n=81)	Indian study (n=97)
Type of NS: FR/SD	66/15	62/35
Gender Male	48(59.2%)	53(54.63%)
Mean age at diagnosis	3.72±2.33	2.8±1.45
Dosage schedule: (2-2.5 g/kg/day)		
Daily dose	33	Single Daily Dose
Alternate day	48	
Cumulative steroid dose		
Pre-Leva	3389.81±2785.22	4109.29±1154
On -Leva	2471.97±2024.98	2491.8±694
Post-Leva	661.37±905.37	660.7±10.7
Relapse Rate		
Pre-Leva (n=81)	3.30 ± 0.46	2.41±0.5
On-Leva (n=57)	0.84± 0.94	1.3±0.7
Post-Leva (n=51)	1.06± 0.99	0.48±0.8
Outcome on Leva (n=57)		
Effective	46(80.70%)	77.3%
Not effective	11(19.29%)	15%

Levamisole has been used in different dosage schedule (either daily single or on alternate day) and in dose range (2-2.5 mg/kg/day) for wide range of duration (12-24 months) in different ethnic populations with more or less similar outcome without significant adverse events.^15^-^20^ During earlier years, levamisole was used as alternate day levamisole 2.5 mg/kg in two divided doses for six months to 18 months but more recently it is being used as 2-2.5 mg/kg as singe alternate day or as daily dose for more than two years.^6^,^15^-^17^,^19^

Ekambram S et al. used leva as daily single dose (2 mg/kg/day) in all children, Fu et al. and Samuel et al. used daily dose leva in children who were non-responders to alternate day dose.^17^,^20^ On the other hand, Abeyagunawardena AS et al. used daily high dose (2.5 mg/kg/day) in those children who failed to alternate (2.5 mg /kg/day) day leva and found better outcome.^15^ Ekambram et al. in a study from our neighboring country (Table-IV) used single daily dose in same age group (Indian vs ours 2.8±1.45 vs ours 3.72±2.33 years) for same duration (18.7±6.4 vs 15.68±9.39) in children with FR and SDNS as we have used in the current study ; and showed that leva was effective in 77.3% of patients which is almost similar to our study (80.7%).Post -leva relapses were higher in our study (1.06±0.99) than in an Indian study (0.48±0.8).^17^ There was reduction in cumulative dose of steroid in children treated with leva (Table-I) which is similar to other studies.^19^,^21^,^22^

Levamisole has been compared recently in RCT with MMF by Sinha A et al. and found that leva and MMF are equally effective (40.8% vs. 34.2%) in maintaining remission and in preventing relapses (14.5% vs. 16.4%) without significant toxicity in children with FR and SDNS.^23^ We also did not find significant adverse effects suggesting safety of drug as observed by others.^20^-^24^ Considering the results of Sinha A et al. in above RCT, Vivarelli M et al. also suggested use levamisole in FR/SD.^25^ Levamisole is also under investigation as adjuvant agent after induction of remission with daily dose in children during first episode of nephrotic syndrome to prevent future relapses and morbidity associated with long term steroid therapy.^14^

### Strength and limitations of this study

It is a retrospective data, lack of equal number of FR and SD, use of different dosage schedule (daily in 33 and alternate day in 48 cases), lack of strict definition of effectiveness which may have led to exclusion of SD cases who were switched to cyclophosphamide/CNI before completion of 6 months of minimum duration, lack of strict monitoring for side effects like anti-nuclear cytoplasmic antibodies (ANCA) and variation of steroid dosage for treatment of relapse which was high during initial years and lower in the later years of practice as reported previously.^12^

The strength of this study is sufficient number of cases, for long duration of follow up by experienced pediatric nephrologist in a tertiary care center, reflecting the variations in personal practice depending upon the available evidence over a period of more than decade from developing country.

## CONCLUSION

We found that levamisole was effective in maintaining remission in 90% while on treatment, whereas remission was maintained after discontinuation in 76.4% cases. We recommend that levamisole may be tried as first line steroid sparing agent before embarking on other immunosuppressive therapies in children with FR/SDNS. However, more studies are needed to provide evidence of the optimal duration, efficacy and optimal dose and its dosage schedule in these children.

### Authors’ Contributions:

**KNM** has major contribution from conceptualization, manuscript writing and preparation, final editing and is responsible for integrity of research.

**HMH:** Literature search and referencing, data collection and write up.

**AMZ:** Literature search, data computation and write up.

**NRV:** Data collection and write up.

All authors have read and approved the final manuscript.
